# Improving Thrombolysis for Acute Ischemic Stroke: The Implementation and Evaluation of a Theory-Based Resource Integration Project in China

**DOI:** 10.5334/ijic.5616

**Published:** 2022-02-08

**Authors:** Qian Fu, Xiaojun Wang, Donglan Zhang, Lu Shi, Wei Wang, Zhangbao Guo, Ping Shan, Guohua Chen, Zhanchun Feng

**Affiliations:** 1 School of Medicine and Health Management, Tongji Medical College, Huazhong University of Science and Technology, Wuhan, Hubei, CN; 2 Wuhan Pulmonary Hospital, Wuhan Institute for Tuberculosis Control, Wuhan, CN; 3 Department of Health Policy and Management, College of Public Health, University of Georgia, US; 4 Department of Public Health Sciences, Clemson University, US; 5 Wuhan No. 1 Hospital, Wuhan, Hubei, CN

**Keywords:** thrombolysis, ischemic stroke, emergency medical services, door-to-needle time

## Abstract

**Introduction:**

Intravenous thrombolysis for acute ischemic stroke remains underused in the developing countries. In 2016, a theory-based resource integration project was initiated at a major stroke center in China. This report describes the implementation process and results of the quality improvement project.

**Description:**

Eighteen environment-tailored interventions were implemented, including stroke code activation, electronic wristband bundling, structured information sharing, etc. The project was implemented from July 2016 to June 2017. A total of 519 acute ischemic stroke patients were included. After the intervention, median DNT decreased from 62 min to 37 min (*P* < 0.001). The percentage of cases treated within 30, 45 or 60 minutes increased from 2.5%, 17.4% and 44.6% to 27.4%, 69.4% and 84.7% respectively (*P* < 0.001). The median length of inpatient stay decreased from 10 days to 8 days (*P* < 0.001). The proportion of patients with severe disability decreased from 25.5% to 15.8% post-intervention.

**Discussion:**

Adequate pre-intervention activities are important conditions for the smooth implementation of the complex service integration initiative. The new treatment pathway has undergone a process of destruction, remodeling and solidification before stable and effective operation. In order to realize the full effect of service integration, whole society efforts are also required.

**Conclusions:**

Introduction of the theory-based resource integration project was associated with increased thrombolysis administrations, shorter DNT, and no statistically significant change in adverse outcomes. The basic principles of this project might be applicable to various resource settings.

## Introduction

Stroke is one of the most common causes of death worldwide and the leading cause of morbidity and mortality in China [1,2]. In eligible patients with acute ischemic stroke (AIS), intravenous thrombolysis is the current gold standard treatment [3]. Clinical outcomes of the therapy, however, are highly time dependent [4,5].

Despite the significant improvement of thrombolysis administration in developed countries, this therapy remains underused in the developing world [6,7]. Evidence from China shows that only 2.5% of AIS patients received intravenous thrombolysis and the door-to-needle times (DNT) in most hospitals significantly exceeded the 60-min benchmark [8,9]. To promote integrated stroke care, the Chinese government initiated the stroke center certification program in 2015. Wuhan No. 1 Hospital is one of the 30 Certificated Demonstration of Advanced Stroke Centers in China. A series of resource integration interventions were implemented from July 2016 to June 2017 to improve thrombolysis for AIS.

Improving thrombolysis for AIS is a well-studied area with more than 20 reported strategies [10]. Most of these strategies were established in developed countries, relying on well-educated communities, excellent emergency service system and sufficient acute stroke treatment resources [11]. Implementation of these strategies in some low-resource setting regions in developing countries might not be applicable. What’s more, advanced hospitals in developing countries are facing more implicit and intractable barriers from social and technical aspects [12]. Some hospitals have begun to introduce new technologies – such as intelligent authentication system or educational robot – to solve these situational problems. However, majority of the existing research on integrated stroke care have been limited to the clinical paradigm [13,14]. Thus led to the scarce of theoretical construction on the integration of emerging information resources and technologies in complex environment.

The latest progress in strategy management suggested that heterogeneous resource integration was the center narrative of service value creation [15]. While the integration of operand resources – such as equipment and facilities – formed the material foundation, the integration of operant resources – including knowledge and information – further services as the key to service quality promotion, according to the theory of service ecosystem [16,17]. We thus hypothesized that the integration of heterogeneous resources could improve thrombolysis use and clinical outcome in patients with acute ischemic stroke.

We established a theoretical framework to guide the resource integration initiatives for intravenous thrombolysis. Members of the acute stroke treatment team at our hospital were surveyed and 18 environment-tailored interventions were implemented, including stroke code activation, electronic wristband bundling, structured information sharing by multimedia instant messenger group (***Table 1***). Here, we present the implementation processes and results of the theory-based resource integration project in Wuhan No. 1 Hospital.

**Table 1:** Elements of the multifaceted resource integration project.


RESOURCE INTEGRATION STRATEGY		INITIAL RECOGNITION AND EMERGENCY TRANSPORTATION STAGE	FAST TRIAGE AND PRELIMINARY DIAGNOSIS STAGE	RAPID IMAGING AND THROMBOLYSIS ADMINISTRATION STAGE

Operand resource integration		Sharing stroke ambulance with emergency center	Setting up neuro emergency	Thrombotic drugs stored in the CT room

Operant resource integration				

	Information-collection	Community education EMS staff training	Training stroke emergency nurses Training emergency triage nurses Using stroke screening tools Using a electronic wristband	Rapid acquisition of brain imaging

	Information-sharing	Pre-notification by EMS	Single-call activation Stroke code activation	Structured information sharing by multimedia instant messenger group

	Decision-making	\	Rapid triage protocol Transport of patients by EMS directly to the scanner	Rapid interpretation of brain imaging Thrombolysis administering in the scanner/imaging area


CT indicates computerized tomography and EMS, emergency medical services.

## Description

### Study setting

Wuhan No. 1 Hospital is established one of the largest stroke centers in Central China serving a population of 31 million within the metropolitan Wuhan area. Before the resource integration project, Wuhan No. 1 Hospital admitted around 900 patients with AIS annually, of these approximately 8.8% received intravenous thrombolysis. A patient with suspected AIS was usually admitted to the separated emergency department (ED), where neurologists could not arrive until at least ten minutes later. Procedures prior to intravenous thrombolysis administration included: National Institutes of Health Stroke Scale (NIHSS) scoring, measurement of vital signs, collection of blood samples, a non-enhanced head CT scan, medical insurance registration and prepayment. After contraindications were excluded, the intravenous thrombolysis would be administered after the patient was moved to the inpatient department of neurology located in another building (Supplementary file 1).

### Intervention

#### Conceptual framework of the intervention

**Figure 1:**
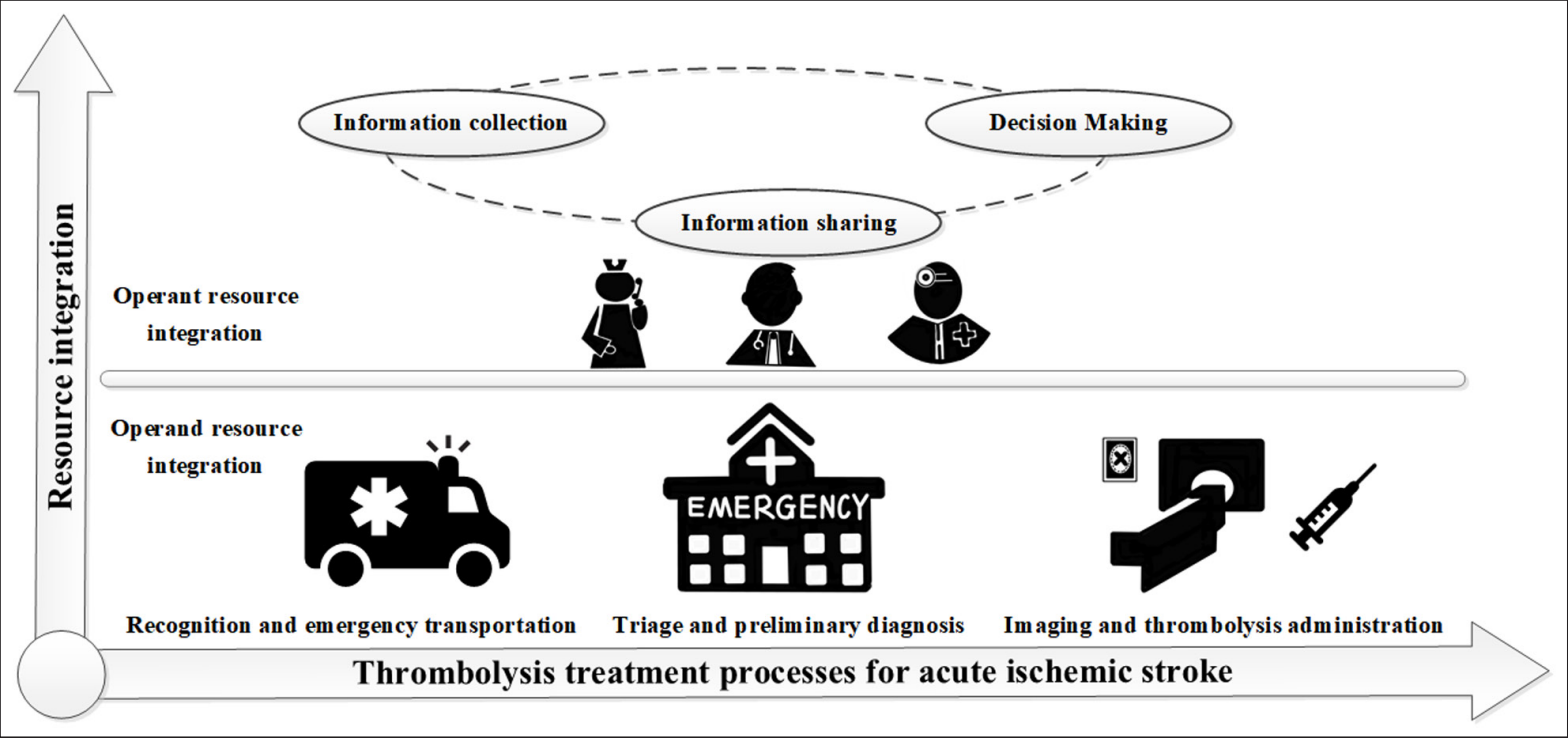
**Conceptual framework of the project.** This framework includes two dimensions composed of resource integration strategies (including operant resource integration and operand resource integration) and intravenous thrombolysis treatment processes (including recognition and emergency transportation stage, triage and preliminary diagnosis, imaging and thrombolysis administration).

A conceptual framework of resource integration for intravenous thrombolysis was developed based on a systematic literature review. This framework included two dimensions composed of the resource integration strategies and the thrombolysis treatment processes for AIS patients (***Figure 1***). The resource integration strategies include operand resource integration and operant resource integration, which was further divided into information collection, information sharing and decision-making. Intravenous thrombolysis process was divided into three stages: the initial recognition and emergency transportation stage (from onset to ED), fast triage and preliminary diagnosis stage (from ED to CT imaging room), the rapid imaging and thrombolysis administration stage (from CT imaging room to thrombolysis administration).

#### Preparation prior to implementing interventions

Stroke center employees were interviewed in a two-round survey. In the first round, two questions were asked in each block of the resource integration matrix: (1) ‘Is there any defect in this segment?’ and (2) ‘Can you suggest any strategy to improve this situation?’. Forty-three members (out of 45) responded to the survey and 23 strategies were suggested. A checklist was then made based on these alternative strategies. In the second round of the investigation, 21 specialists – from disciplines including neurology, emergency care and imaging – were asked to evaluate the importance and feasibility of these strategies on the checklist. Eighteen resource integration intervention strategies were finally selected after a series of discussions based on the results of the investigation (***Table 1***).

In order to implement these complex interventions smoothly, we made preparations mainly from the following three aspects. 1) Relevant departments were organized to communicate repeatedly on the implementation plan under the leadership of the hospital director; 2) New processes, specifications and tools were developed and imparted by special trainings before the intervention; 3) The economic incentive scheme – based on the quantity and quality of thrombolysis administration – was established.

#### Initial recognition and emergency transportation stage

Community education, emergency medical service (EMS) staff training and ambulance sharing were implemented to promote the early identification and timely transportation of stroke patients. Stroke quick identification cards, “Stroke 1–2–0” (Chinese version of FAST), were distributed in communities during each educational activity [18,19]. An educational program for the EMS staff was implemented to make sure that the EMS dispatchers assign possible stroke patients the highest priority level. This program included instructions to recognize stroke symptoms and emphasis on the importance of rapid treatment. A 24/7 ambulance, equipped with neurologists and stroke emergency department nurses, was shared with the Wuhan emergency dispatching network. The emergency information platform of Wuhan emergency center and Wuhan NO. 1 Hospital was connected for the prenotification by EMS.

#### Fast triage and preliminary diagnosis stage

An independent neurology emergency room with 24-hour neurologists and emergency nurses was set up in the emergency area. Four stroke emergency nurses were trained to be the “integrator” of acute stroke treatment resources. They had the responsibility to participate in all the intravenous thrombolysis treatment processes, including patient registration, stroke green channel initiation, rapid blood sample collection, CT room inspection accompaniment, mixing of alteplase and docking with the inpatient department. Emergency triage nurses of the hospital were trained to get familiar with the early recognition of stroke.

The following changes were made to the triage and preliminary diagnosis protocol: use of a stroke code, single-call activation, application of an electronic patient wristband, rapid triage and transport of patients by EMS directly to the CT scanner. The stroke code was used to facilitate the patient pre-registration, post treatment payment qualification and advanced order of laboratory tests and CT scanner. The stroke code would be activated by the stroke emergency nurse once a stroke patient was evaluated within the therapeutic window for intravenous thrombolysis. We used an electronic patient wristband system to facilitate the recording of treatment time nodes and the improvement of service process. (Supplementary file 2) The stroke patients, after rapid assessment for medical stability and neurological signs, could be directly transported to the CT scanner on the EMS stretcher.

#### Rapid imaging and thrombolysis administration stage

**Figure 2:**
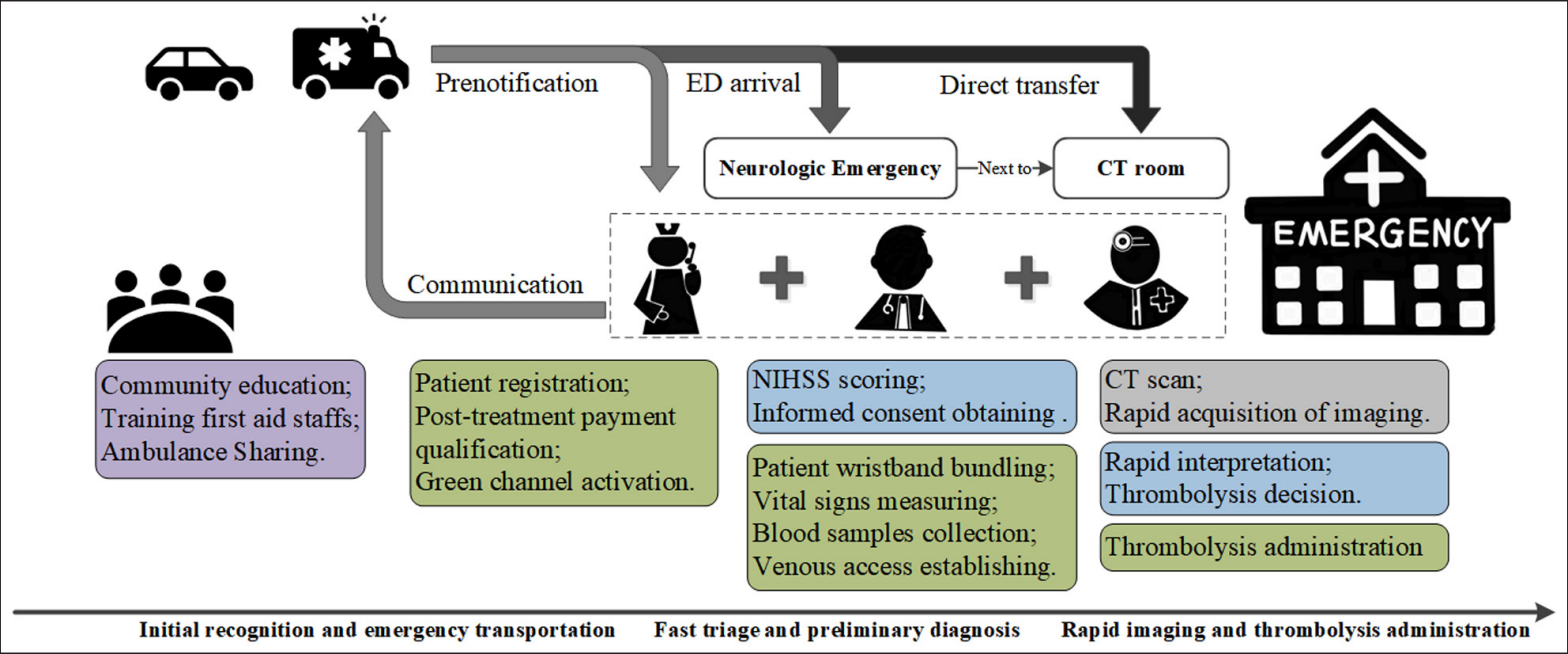
**Flowchart of the new treatment process.** The new treatment process is designed in parallel. Tasks in different stages are clearly assigned to each of the members of the acute stroke treatment team.

Strategies to reduce the time during brain imaging and thrombolysis administration included rapid acquisition and interpretation of brain imaging, structured information sharing by multimedia instant messenger group and administering alteplase in the CT-room. The CT imaging protocol of stroke patients was simplified based on the initial diagnosis of the patients. After the acquisition of the CT-scan, the radiologist, together with a neurologist, interpret the CT-images immediately at the monitor. For all patients eligible for thrombolysis administration, immediate mixing and administration of alteplase on the scanner table or just outside the CT suite was required (***Figure 2***).

#### Thrombolysis registry and study population

A local thrombolysis registry was established in January 2015. All suspected AIS patients having received intravenous thrombolysis were prospectively included. The registry contains multiple variables including basic patient demographics, medical comorbidities (hypertension, diabetes mellitus, atrial fibrillation, coronary disease, prior stroke), smoking status, National Institutes of Health Stroke Scale (NIHSS) score on admission, mode of emergency department (ED) arrival, in-hospital mortality, length of inpatient stay, hospitalization expenses and modified Rankin scale (mRS) at 90 days. The relevant time points, including symptom onset, arrival at the emergency room and intravenous thrombolysis administration were recorded. Data was collected with a registration form filled by nurses and neurologists. Recorded data was overseen by a dedicated neurologist.

Data for the present study were extracted from the electronic medical record and quality control database from January 2015 to June 2019. Cases were included for analysis under the following criteria: 1) age above 18 years; 2) received intravenous thrombolysis treatment within 6 hours of last seen well. In-hospital stroke patients and transferred patients were excluded.

### Evaluation

The primary outcome was continuous and categorical DNT for intravenous thrombolysis. The secondary outcomes were median OTD times, thrombolysis administration rate, average length of inpatient stay, average inpatient medical expenses, mRS at 90 days, and rate of symptomatic intracranial hemorrhage. A repeated CT scan was performed 48 hours from presentation and hemorrhage associated with at least a 4-point increase in NIHSS would be determined symptomatic intracerebral hemorrhage. The percentage of stroke mimics was included as a balancing measure.

July 2016 was set as the cut-off point of pre and post intervention. The one year implementation period was included in the post-intervention stage. We used Statistical Process Control charts as the primary means of analysis for DNT to assess its evolutionary trend after implementation. Continuous variables were reported in mean (Standard deviation, SD) or median (Interquartile Range, IQR), and categorical variables were reported in absolute and relative frequencies. Univariate analyses comparing subjects in the pre-intervention and post-intervention periods were performed with *t* test or a Kruskal–Wallis nonparametric test for continuous variables and *χ^2^* test for categorical variables.

Multivariable logistic regression models were used to estimate the association between the interventions and categorical DNT or mRS at 90 days. All multivariable models adjusted for age, sex, baseline NIHSS score, history of hypertension, diabetes mellitus, atrial fibrillation, coronary heart disease, and prior stroke history.

Statistical significance was set at *p* < 0.05 (two-sided). Statistical analyses were performed with SAS 9.4 and JMP 13 (SAS Institute Inc., Cary, NC, USA).

## Ethical Approval

Ethics committee approval and patients’ written informed consent were not required because this was a quality improvement study that involved assessment of the standard of care and only de-identified information from routine hospital data is used.

## Result

A total of 519 AIS patients were treated with intravenous thrombolysis from January 2015 to June 2019. Of these, 121 patients were treated during the pre-intervention period (from January 2015 to June 2016) and 398 patients were treated during the post-intervention period (from July 2016 to June 2019). On average, there were 72 stroke alerts monthly in the pre-intervention period compared with 82 stroke alerts monthly in the post-intervention period. The rates of intravenous thrombolysis administration were 9.4% (121 cases/1295 stroke alerts) and 13.6% (398 cases/2938 stroke alerts; *P* < 0.001), respectively. There was no significant difference between patients treated pre-intervention and post-intervention by demographics, baseline NIHSS, mode of ED arrival and comorbidities (***Table 2***).

**Table 2:** Characteristics of Patients in Pre-intervention and Post-intervention.


	PRE-INTERVENTION (*N* = 121)	POST-INTERVENTION (*N* = 398)	*P* VALUE

Age (y), (mean ± SD)	67.6 ± 11.4	66.2 ± 11.9	0.240

Female (%)	46 (38.0)	143 (35.9)	0.676

Transferred by EMS (%)	35 (28.9)	131 (32.9)	0.410

Baseline NIHSS, median (IQR)	4 (3–11)	4 (2-10)	0.080

Prior Stroke/TIA (%)	21 (17.4)	82 (20.6)	0.433

Hypertension (%)	72 (59.5)	211 (53.0)	0.209

Diabetes mellitus (%)	26 (21.5)	110 (27.6)	0.178

Atrial fibrillation (%)	17 (14.0)	55 (13.8)	0.949

Coronary heart disease (%)	20 (16.5)	49 (12.3)	0.231

Current Smoker (%)	63 (52.1)	192 (48.2)	0.461


*P* values are from *t* test or a Kruskal–Wallis nonparametric test for continuous variables and *χ^2^* test for categorical variables. IQR, interquartile range; NIHSS, National Institutes of Health Stroke Scale; and TIA, transient ischemic attack.

**Figure 3:**
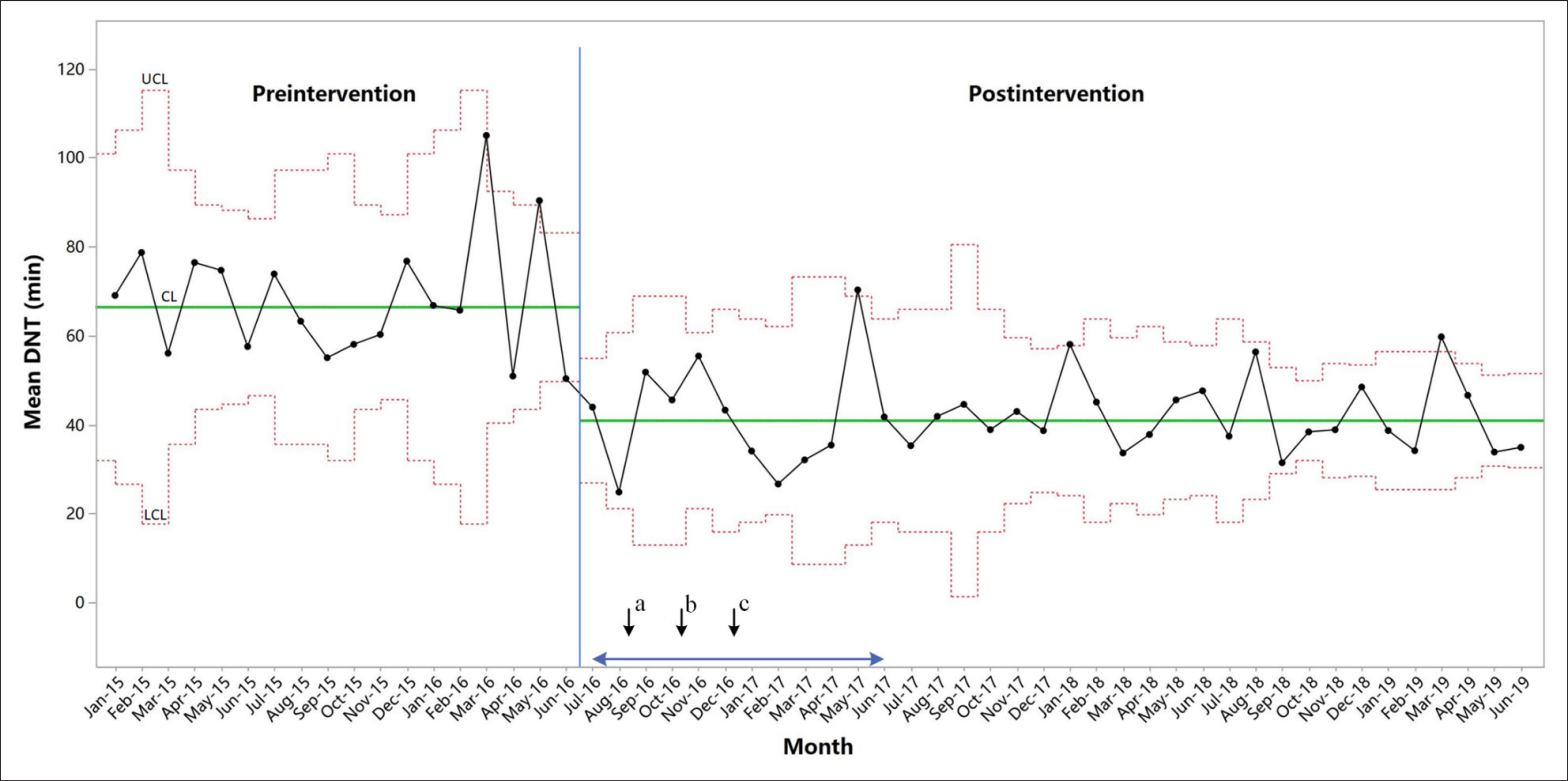
**Statistical Process Control chart of monthly average door-to-needle times.** Blue double arrow line shows the timing of the intervention. Black arrows show the timing of interventions: a. Ambulance sharing, neuro emergency establishing, stroke emergency and emergency triage nurse training, screening tool using, single-call activation, stroke code activation, rapid acquisition of brain imaging, thrombotic drugs storing in the CT room, transport of patients by EMS directly to the scanner, thrombolysis administering in the imaging area were implemented in July; b. Pre-notification by EMS, electronic wristband, rapid triage protocol, rapid interpretation of brain imaging were implemented in September; c. Community education, EMS staff training, structured information sharing by multimedia instant messenger group were implemented in November. DNT, door-to-needle times; CL, center line; LCL, lower control limit; UCL, upper control limit.

Both continuous and categorical DNT decreased significantly in the post-intervention period. Median DNT reduced from 62 (IQR 52–80) min to 37 (IQR 29–48) min (*P* < 0.001) and mean DNT reduced from 67 ± 25 min to 41 ± 19 min (*P* < 0.001). The X-bar chart shows an immediate shift of monthly mean DNT post-intervention (***Figure 3***). The interval of mean DNT gradually narrowed throughout the post-intervention period. The percentage of cases treated within 30, 45 or 60 minutes increased from 2.5%, 17.4% and 44.6% to 27.4%, 69.4% and 84.7% respectively (*P* < 0.001; ***Table 3***).

**Table 3:** Time Between ED Arrival and Thrombolysis Administration (Categorical Door-to-Needle Time in Minutes).


	PREINTERVENTION (*N* = 121)	POSTINTERVENTION (*N* = 398)	UNADJUSTED ODDS RATIO	*P* VALUE	ADJUSTED ODDS RATIO	*P* VALUE

<60 min (%)	54 (44.6)	337 (84.7)	6.85 (4.37–10.75)	<0.001	7.31 (4.59–11.63)	<0.001

<45 min (%)	21 (17.4)	276 (69.4)	10.77 (6.43–18.05)	<0.001	11.93 (7.04–20.20)	<0.001

<30 min (%)	3 (2.5)	109 (27.4)	14.84 (4.62–47.65)	<0.001	14.84 (4.62–47.65)	<0.001


Multivariable models include adjustment for age, sex, medical insurance, baseline NIHSS score, history of hypertension, diabetes mellitus, atrial fibrillation, coronary heart disease, prior stroke and current smoker.

Prevalence of fatal intracranial hemorrhage was 1.7% (n = 2) pre-intervention and 1.3% (n = 5) post-intervention (*P* = 0.740). The proportion of stroke mimics was 5.8% (n = 7) pre-intervention and 4.5% (n = 18) post-intervention (*P* = 0.389). A sensitivity analysis was performed on median DNT removing the stroke mimics and the results remained significant, reduced from 62 (IQR 52–80) min to 37 (IQR 29–46) min (*P* < 0.001).

There was a decrease in the median length of inpatient stay from 10 (IQR 8–16) days to 8 (IQR 6–11) days (*P* < 0.001). No significant difference was detected between the periods in the inpatient medical expenses (48422 ± 62664 versus 42766 ± 54563 post-intervention; *P* = 0.340) or median OTD times, 120 (IQR 90–180) min versus 115 (IQR 89–155) min post-intervention (*P* = 0.125).

For 90-day mRS scores, stroke mimics and missing data (n = 15 pre-intervention, n = 87 post-intervention) were excluded. After the intervention, significant improvement was shown only in patients with severe disability (***Table 4***). The proportion of patients with severe disability decreased from 25.5% pre-intervention to 15.8% post-intervention.

**Table 4:** Patient outcome (mRs at 90 days).


	PREINTERVENTION (*N* = 106)	POSTINTERVENTION (*N* = 311)	UNADJUSTED ODDS RATIO	*P* VALUE	ADJUSTED ODDS RATIO	*P* VALUE

mRs 0–1 (%)	57 (53.8)	183 (58.8)	1.12 (0.59-1.85)	0.326	1.27 (0.78–2.31)	0.471

mRs 2–3 (%)	17 (16.0)	68 (21.9)	1.44 (0.83-2.06)	0.074	1.35 (0.92–1.71)	0.183

mRs 4–5 (%)	27 (25.5)	49 (15.8)	1.83 (1.22-5.47)	0.029	1.62 (1.36–7.64)	0.037

Mortality (%)	5 (4.7)	11 (3.5)	1.13 (0.57-2.10)	0.629	0.94 (0.58–2.82)	0.562


Multivariable models include adjustment for age, sex, medical insurance, baseline NIHSS score, history of hypertension, diabetes mellitus, atrial fibrillation, coronary heart disease, prior stroke and current smoker.

## Discussion

With implementation of the theory-based resource integration project, we achieved a substantial reduction in median DNT from 62 to 37 min among AIS patients. A continuous gain in thrombolysis administration was observed at 90 days, with a reduction of proportion of patients with severe disability from 25.5% to 15.8% post-intervention. Thrombolysis administration rate in our center increased from 9.4% to 13.6% post-intervention without significant changes in the symptomatic intracerebral hemorrhage rate and proportion of stroke mimics.

In our study, the median DNT reduced immediately after the intervention in July 2016. Both the extent and the timing of DNT reductions exceeded our expectations. This might be partly due to the intensive pre-intervention communications, through which the center members had reached a high degree of consensus on the goals and strategies of the project. Another important factor was the launching of supporting institutions. A series of administrative orders and incentives were carried out in advance, aiming to motivate the collaboration between different departments of the stroke center. Since the interventions were implemented in a one-year time span, a continuous reduction in DNT was also expected. However, the mean and median DNT had remained stable since the significant decline in July 2016. Meanwhile, it was noted that the interval of mean DNT gradually narrowed in the post-intervention period (***Figure 3***). Accordingly, the proportion of patients treated within 30, 45 or 60 min increased with each passing year since 2016. This might indicate that the new treatment pathway was undergoing a process of solidification, which was consistent with the theory of service ecosystem. Service innovation needs to go through a process of destruction, remodeling and solidification [20].

Given the interventions targeting the initial recognition and emergency transportation stage, we would expect a reduction in OTD times. However, there was no significant difference in the median OTD times between the two periods. Potential reason for this result might be the fact that the EMS utilization behavior of AIS patients barely changed during the intervention. Acute stroke patients in China are more likely to arrive at the hospital by private vehicles or public transportations, rather than calling the EMS. A recent analysis from the Chinese Stroke Center Alliance showed that only 12.5% (69 841/560 447) of the acute stroke patients were transported by EMS [21]. The proportion of patients brought in by ambulance in our study was 28.9% pre-intervention and 32.9% post intervention, much lower than that in developed countries, which might limit the effectiveness of the prenotification intervention [22,23]. Therefore, whole society efforts are needed to make stroke patients and their families aware of the importance of calling EMS in time [24].

Implementation of the project was followed by a significant increase in thrombolysis administration rate (from 9.4% to 13.6%) and sustained decrease in the median length of hospital stay (from 10 days to 8 days). Given that the amount of acute stroke treatment resources barely changed during the intervention in our center, it is likely that the utilization of resources has been significantly improved. Nevertheless, the length of stay of AIS patients in China is still much longer than that in the USA and other countries [25]. This should be interpreted in the context that China has not yet built a complete rehabilitation system for stroke patients, so that a longer inpatient stay is necessary for better rehabilitation [26,27]. In addition, the reduction in hospital stay was not accompanied with a decrease in hospitalization expense, indicating the effect of the intervention was more reflected in the utilization of hospital resources, rather than the reduction of the economic burden. However, a significant improvement in patient outcome was detected that fewer patients had severe disability 90 days after the intervention. Even though, there was no significant reduction in mortality, which should be interpreted in the context of an overall low mortality in the first 3 months [28].

After more than 20 years since the results of the first randomized controlled trial suggested efficacy of intravenous thrombolysis in AIS patients, there is still a huge gap in the application of intravenous thrombolysis around the world [29,30]. Recent reports of longitudinal studies showed substantial decrease of DNT to less than 30 minutes in the hospitals of United States, Finland, Austria and Norway with high thrombolysis administration rates (from 15.3% to 16.8%) [28,31,32,33]. However, intravenous thrombolysis administration in developing countries is still underused with administration rates from 2.5% to 5.3% and median DNT from 63 to 95 min [9,12,34]. The main reasons for this gap are considered to be the different resource setting and social environment [35]. Acute stroke treatment resources in developing countries are largely unevenly distributed and lack of cooperation [34]. Thus the integration of resources, especially operant resources such as information and knowledge, has to overcome serious barriers from infrastructure to social institutions [36,37]. For example, an AIS patient in China might experience serious treatment delays due to the time-consuming pretreatment medical insurance certification or just because the accompanying family could not understand the benefits of intravenous thrombolysis [38]. As a result, there is an urgent need to establish more flexible resource integration principles for intravenous thrombolysis for developing countries.

An important difference between this intervention and several others is the use of a theory-based resource integration framework. Several strategies were specially adjusted to the local environment according to the framework, such as the post treatment payment qualification by using a stroke code, the application of an electronic wristband to automatically record the time points. From this point of view, various environment-adapted strategies that can improve the quality and efficiency of information collection or sharing might be potential services improvement opportunities. The basic principles of this framework might be adapted to various resource settings and is conducive to the continuous improvement of intravenous thrombolysis. For example, the application of the telemedicine-based assessment on ambulance or even a simple text messaging could save the time of diagnosis by improving the efficiency of information sharing, in which the later is also feasible in many low resource setting areas [39,40].

Our study has several limitations. First, it is a single-center study and unknown contextual factors might limit generalizability. Second, inferences regarding cause and effect will be limited by the pre–post study design due to confounding factors that we might not be able to adjust for. Third, we implemented the interventions concurrently rather than testing the effects of each intervention in a controlled setting. Forth, we included the one-year intervention process in the post-intervention stage, which may lead to underestimation of the effect. However, it is not our intention to demonstrate the effectiveness of each strategy, but to comprehensively present the process and result of the theory-based resource integration project for broader reference.

## Lessons Learned

- Adequate pre-intervention activities are important conditions for the smooth implementation of a complex service integration initiative.
- The integration of heterogeneous resources has to overcome serious barriers from infrastructure to social institutions.
- A new treatment pathway might undergo a process of destruction, remodeling and solidification before stable and effective operation.
- Various environment-adapted strategies that can improve the quality and efficiency of information collection or sharing might be potential services improvement opportunities.

## Conclusion

Introduction of the multifaceted resource integration project was associated with increased thrombolysis administrations and significantly shorter DNT, with no significant changes in the symptomatic intracerebral hemorrhage rate and proportion of stroke mimics. The basic principles of this project might be adapted to various resource settings. Future studies with experimental or quasi-experimental designs are needed to better assess the effectiveness of the resource integration framework.

## Additional Files

The additional files for this article can be found as follows:

10.5334/ijic.5616.s1 Supplementary file 1. Treatment process before intervention.

10.5334/ijic.5616.s2 Supplementary file 1. Introduction on the electronic patient wristband system.
