# Supplementary figures and images for: Improving Thrombolysis for Acute Ischemic Stroke: The Implementation and Evaluation of a Theory-Based Resource Integration Project in China
Source: Int J Integr Care. 2022 Feb 8;22(1):9. doi: 10.5334/ijic.5616 (PMC8833266; doi:10.5334/ijic.5616)

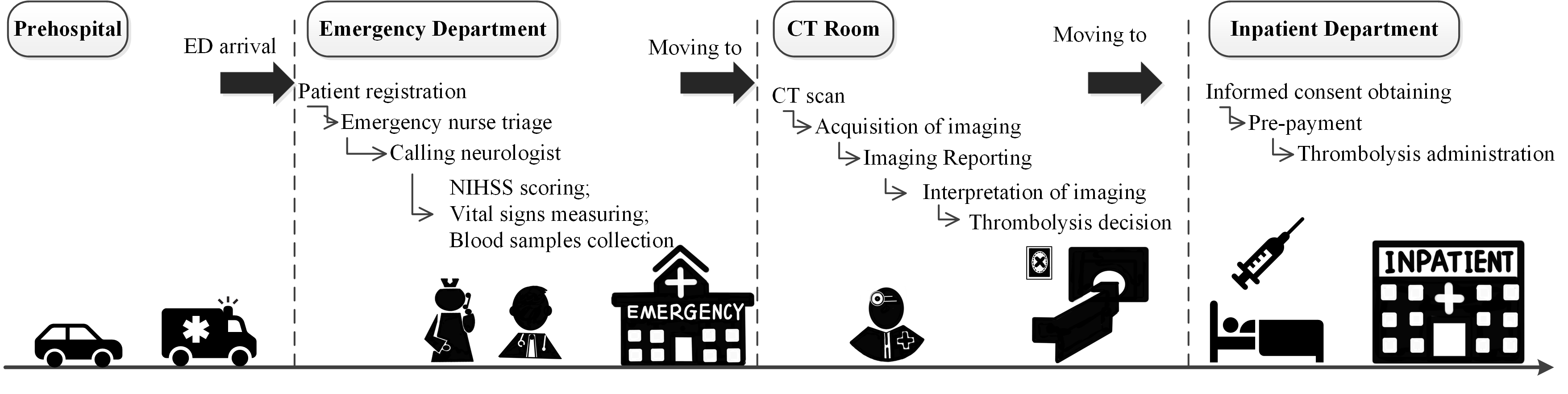

Supplement: Supplementary file 1. — Treatment process before intervention. [file ijic-22-1-5616-s1.tif]
