# Supplementary material for: Improving Thrombolysis for Acute Ischemic Stroke: The Implementation and Evaluation of a Theory-Based Resource Integration Project in China
Source: Int J Integr Care. 2022 Feb 8;22(1):9. doi: 10.5334/ijic.5616 (PMC8833266; doi:10.5334/ijic.5616)
Supplement: Supplementary file 1. — Introduction on the electronic patient wristband system. [file ijic-22-1-5616-s2.pdf]

Introduction on the electronic patient wristband system

I. System component

The electronic patient wristband system mainly consist of 4 parts, including electronic patient wristbands, NFC punch buttons, Bluetooth location punch recording device, data acquisition and analysis software.

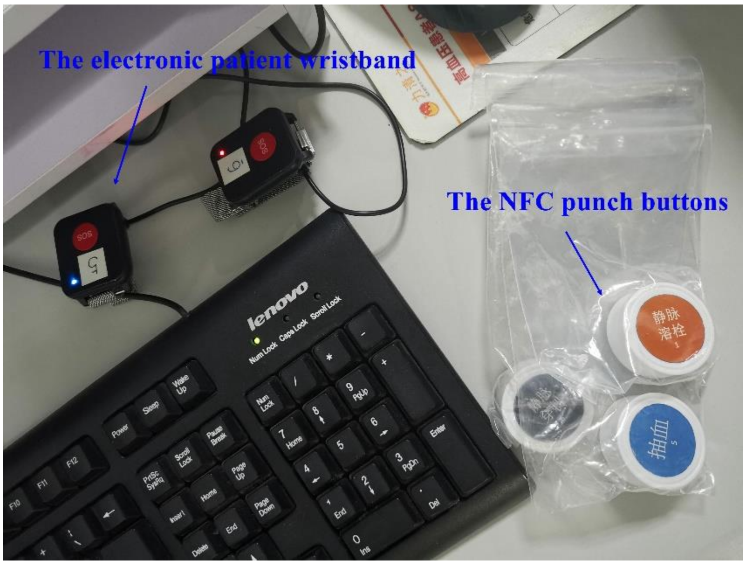

II. System operation

An electronic wristband would be put on the patient's wrist when he arrives. The electronic patient wristband has built-in Bluetooth and NFC modules. Position time nodes such as arrival at the CT room would be automatically recorded by the Bluetooth location punch recording device pre-installed. Treatment time nodes would be recorded by the stroke nurse by touching the wristband with different NFC buttons.

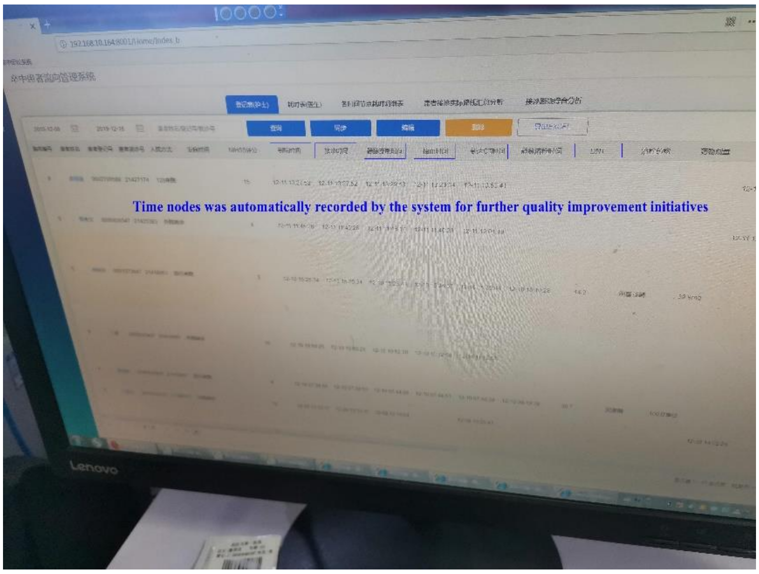

III. System advantages

In the old treatment process, stroke nurses need to manually record the various time points of treatment, including symptom onset, arrival at the emergency room and intravenous thrombolysis administration, etc. There are two defects in this process: 1) Manual recording delays valuable patient treatment time; 2) When it is difficult to record in time due to emergency, recall bias exists in the subsequent recording.

In the new process, a Bluetooth patient wristband is used to facilitate the recording of treatment time nodes in two approaches. 1) The time nodes when the patient arrived at the emergency room and CT room will be automatically recorded by the system. 2) During the treatment process, the stroke nurse can record the time nodes by touching the wristband with different buttons. All time nodes information and patient information will be automatically collected and sorted by the supporting system for service quality improvement.
